# Clinical Outcomes of Immunocompromised Adults Hospitalized with Pneumococcal Pneumonia: A Case-Control Study

**DOI:** 10.3390/microorganisms9081746

**Published:** 2021-08-16

**Authors:** Julio Ramirez, Thomas Chandler, Stephen Furmanek, Forest Arnold, Jose Bordon

**Affiliations:** 1 Division of Infectious Diseases, Department of Medicine, School of Medicine, University of Louisville, Louisville, KY 40202, USA; thomas.chandler@louisville.edu (T.C.); stephen.furmanek@louisville.edu (S.F.); f.arnold@louisville.edu (F.A.); jbordon@dc-whi.org (J.B.); 2 Washington Health Institute, Washington, DC 20017, USA; 3 Department of Medicine, George Washington University Medical Center, Washington, DC 20052, USA

**Keywords:** immunocompromised host, streptococcus pneumoniae, community-acquired pneumonia

## Abstract

*S. pneumoniae* is a primary etiologic agent of CAP in immunocompromised adults (ICA). Data on clinical outcomes of ICA hospitalized with pneumococcal pneumonia (PP) is limited. The objectives of this study were (1) to define clinical presentation and outcomes of ICA hospitalized with PP and (2) to compare the data to non-immunocompromised adults (non-ICA) hospitalized with PP. This was a case–control study of ICA hospitalized with PP (cases) and non-ICA hospitalized with PP (controls). Data were collected on clinical presentation, treatment, and outcomes. Evaluated clinical outcomes included time to clinical stability (TCS), length of hospitalization (LOH), clinical failure (CF), cardiovascular events (CE), and in-hospital mortality (IHM). One ICA was matched to two non-ICA through propensity score matching. A total of 93 ICA hospitalized with PP and 186 non-ICA hospitalized with PP were evaluated. Antibiotic therapy was appropriate in all patients. Clinical outcomes for ICA versus non-ICA were as follows: TCS 2 days vs. 2 days (*p* = 0.392); LOH 5 days vs. 5 days (*p* = 0.067); CF 4% vs. 6% (*p* = 0.618); CE 10% vs. 6% (*p* = 0.375); and IHM 5% vs. 3% (*p* = 0.296). In hospitalized patients with PP who are treated with appropriate antibiotic therapy, the presence of an abnormal immune system does influence clinical outcomes.

## 1. Introduction

Immunocompromised patients are at high risk for development of community-acquired pneumonia (CAP). In this population, CAP may be due to opportunistic pathogens (e.g., *Pneumocystis jirovecii*), as well as the traditional typical and atypical pathogens causing CAP in non-immunocompromised patients [1].

*Streptococcus pneumoniae* is considered the primary etiology of CAP in immunocompetent as well as immunocompromised adults. It is accepted that immunocompromised patients are at high risk for development of *Streptococcus pneumoniae* CAP, but data on the clinical outcomes of these patients once they acquired pneumococcal pneumonia is limited [2,3]. It can be speculated that in immunocompromised patients with pneumococcal pneumonia, the presence of a compromised immune system may negatively impact clinical outcomes. On the other hand, once patients are on appropriate antibiotic therapy, a compromised immune system may not play a significant role in the patient’s recovery and may not impact clinical outcomes.

With the goal to define the influence of immunosuppression on outcomes of patients with pneumococcal pneumonia, we compared clinical outcomes in immunocompromised and non-immunocompromised adults hospitalized with pneumococcal pneumonia.

## 2. Materials and Methods

### 2.1. Study Design and Patient Population

This was a retrospective case–control study of hospitalized adult (age ≥18 years) patients with pneumococcal pneumonia. The clinical outcomes of immunocompromised adults with pneumococcal pneumonia (cases) were compared to the clinical outcomes of non-immunocompromised adults with pneumococcal pneumonia (controls). Patients were eligible if they were hospitalized between 1 June 2014 and 31 May 2016 at any of the nine adult acute-care hospitals in Louisville, KY.

### 2.2. Study Definitions

A patient was defined as having CAP when the following three criteria were met: (1) presence of a new pulmonary infiltrate on chest radiograph and/or chest computerized tomography scan at time of hospitalization; (2) at least one of the following (a) new cough or increased cough or sputum production, (b) fever >37.8 °C (100.0 °F) or hypothermia <35.6 °C (96.0 °F), or (c) changes in leukocyte count (leukocytosis >11,000 cells/mm^3^, left shift > 10% band forms/microliter, or leukopenia <4000 cells/mm^3^); and (3) no alternative diagnosis at time of hospital discharge that justified the presence of criteria 1 and 2 [4].

A patient with CAP was defined as having pneumococcal pneumonia if a urinary antigen test or blood culture was positive for *Streptococcus pneumoniae.*

Appropriate antibiotic therapy was defined as receiving initial empiric therapy with coverage for *S. pneumoniae*.

Immunocompromised patients were identified by the presence of any of the following medical conditions or treatments that compromise immune function [1]:

(A) Medical conditions: (1) primary immunodeficiency diseases; (2) advanced-stage cancer (stage III or IV cancer or hematologic cancers); (3) advanced HIV infection (CD4 T-lymphocyte count <200 cells/mL or <14%); (4) solid organ transplantation; and (5) hematopoietic stem cell transplantation.

(B) Treatments: (1) receiving cancer chemotherapy; (2) receiving biological immune modulators; (3) receiving corticosteroid therapy with a dose 20 mg prednisone or equivalent daily for at least 14 days prior to hospitalization; (4) receiving disease-modifying antirheumatic drugs (DMARDs).

### 2.3. Study Outcomes

(A) Time to clinical stability was calculated as the number of days from the date of admission to the date that the patient met clinical stability criteria, up to 8 days. Clinical stability was defined as follows: improved clinical signs (improved cough and shortness of breath), lack of fever for at least 8 h, improving leukocytosis (decreased at least 10% from the previous day), and tolerating oral intake. These criteria were recommended by the American Thoracic Society in 2001 and are clinically equivalent to the 2019 recommendations [5].

(B) Length of hospitalization was calculated as the number of days from the date of admission to the date of discharge. Length of hospitalization was right truncated at 14 days in an effort to capture only CAP-related length of hospitalization.

(C) Clinical failure was defined as the need for invasive ventilation, non-invasive ventilation, or vasopressors after the first day of hospitalization.

(D) Cardiovascular events during hospitalization were defined as the development of any of the following: (1) acute myocardial infarction, (2) new arrhythmia, (3) acute worsening of long-term arrhythmia, (4) pulmonary embolism, (5) pulmonary edema, or (6) cerebrovascular accident.

(E) In-hospital mortality evaluated at any time during hospitalization.

### 2.4. Data Collection and Statistical Analysis

Demographic information, clinical data on hospital admissions, microbiological studies, in-hospital treatment, and clinical outcome data were collected. The severity of pneumonia on admission was evaluated by the pneumonia severity index (PSI) and CURB-65 scores [6,7].

Propensity score matching was used to account for differences in baseline characteristics between immunocompromised and non-immunocompromised patients, with immunocompromised patients serving as “treated” subjects. The following variables were used to calculate the probability of being an immunocompromised: age, sex, race, nursing home residence, smoking status, obesity, diabetes, renal disease, congestive heart failure, coronary artery disease, history of stroke, non-cirrhotic liver disease, cirrhosis, hypertension, hyperlipidemia, prior myocardial infarction, atrial fibrillation, hospitalization in the prior 90 days, IV antibiotics in the prior 90 days, chronic dialysis, oral antibiotics in the past 30 days, and chronic antecedent use of aspirin. Patients were matched at a ratio of 1 case: 2 controls by a nearest-neighbor matching algorithm [8,9].

Continuous variables were summarized as medians and interquartile ranges (IQRs). Dichotomous variables were summarized as frequencies and percentages. Standardized mean differences (SMDs) after matching were reported to quantify effect sizes between ICA and non-ICA. Variables with SMDs greater than 0.1 after matching were considered to be potentially unbalanced. Adjusted analysis included conditional logistic regressions and stratified Cox-proportional hazards models to account for matching between groups and were reported as conditional odds ratios (cOR) and stratified hazards ratios (sHR) with 95% confidence intervals (CIs). Presence of co-infection, as well as any variables with an SMD greater than 0.1, were used as additional adjusting variables in regression analysis.

A sensitivity analysis, excluding patients with solid advanced cancer but no chemotherapy or other immunocompromising conditions, as well as their non-immunocompromised matches, was performed for the five outcome variables.

*p*-values less than 0.05 were considered statistically significant. R version 3.5.1 was used for analysis [10].

This study was approved by the Institutional Review Board (IRB) at the University of Louisville Human Subjects Research Protection Program Office (IRB number 11.0613) and by the research offices at each participating hospital. The study was exempt from informed consent.

## 3. Results

### 3.1. Patient Population

A total of 93 immunocompromised adults hospitalized with pneumococcal pneumonia were included in analysis and matched with 186 non-immunocompromised adult controls. Patient characteristics are described in Table 1. Median age for immunocompromised patients was 63 (IQR: 56–72) years and 61 (IQR: 55–73) years for non-immunocompromised patients. Both groups were majority male, with 48 (52%) immunocompromised patients and 101 (54%) non-immunocompromised patients. The most prevalent comorbidities in each group were hypertension (60% immunocompromised vs. 62% non-immunocompromised), former smoking status (43% immunocompromised vs. 40% non-immunocompromised), and hyperlipidemia (34% immunocompromised vs. 33% non-immunocompromised). For immunocompromised patients, 32% were hospitalized for more than two days in the past 90 days, versus 28% of non-immunocompromised. No variables had an SMD greater than 0.1.

**Figure 1:**
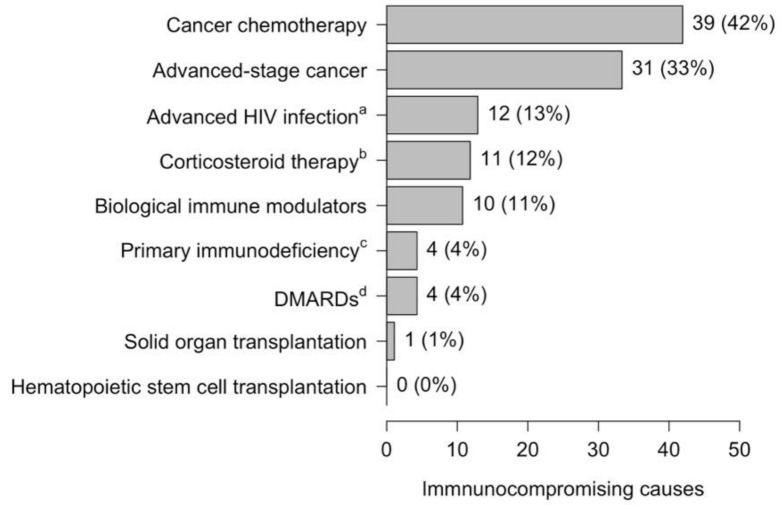
Causes of immunocompromising condition among patients hospitalized with pneumococcal pneumonia. ^a^ HIV infection present with CD4 count < 200 or CD4 percent < 14%; ^b^ Corticosteroid use consisted of a dose of 20 mg prednisone or equivalent daily for at least 14 days; ^c^ Primary immunodeficiency included asplenia and B-cell immunodeficiencies; ^d^ Disease-modifying antirheumatic drugs included cyclosporin, 13-cyclophosphamide, hydroxychloroquine, and methotrexate.

The distribution of immunocompromising causes is depicted in Figure 1. The most frequent causes were cancer-related, with cancer chemotherapy in 39 (42%) patients and late-stage cancer in 31 (33%) patients. Lung cancer in 21 (23%) patients, leukemia in 7 (8%) patients, head or neck cancers in 7 (8%) patients, colorectal cancer in 6 (6%) patients, and breast cancer in 3 (3%) patients were the most frequent cancers responsible for immunocompromised state, either through advanced cancer or chemotherapy. A total of four immunocompromised patients had neutrophil counts of less than 1000 per µL.

Vaccination history was available for 50 (54%) immunocompromised patients and 95 (51%) non-immunocompromised patients. Of these, 11 (22%) immunocompromised patients and 20 (21%) non-immunocompromised patients had received either the conjugate or polysaccharide vaccine.

In regard to the most common symptoms for CAP, cough and/or shortness of breath were documented in 85 (91%) immunocompromised patients and 178 (96%) non-immunocompromised patients.

### 3.2. Microbiological Findings and Antibiotic Treatments

All patients had urinary antigen testing for *S. pneumoniae*. Blood cultures were performed in 87 (94%) immunocompromised patients and 186 (97%) non-immunocompromised patients. Of these, 4 (5%) immunocompromised and 26 (14%) non-immunocompromised patients had positive blood cultures for *S. pneumoniae*.

Identified co-infections of another microorganism besides *S. pneumoniae* were present in 14 (15%) immunocompromised patients and 28 (15%) non-immunocompromised patients. Immunocompromised patients were most commonly co-infected with methicillin-resistant *Staphylococcus aureus* (*n* = 3, 3%), *Haemophilus influenzae* (*n* = 2, 2%), and human metapneumovirus (*n* = 2, 2%). Among non-immunocompromised patients, the most commonly identified co-infections were rhinovirus/enterovirus (*n* = 6, 3%), *Haemophilus influenzae* (*n* = 3, 2%), methicillin-resistant *Staphylococcus aureus* (*n* = 3, 2%), influenza A (*n* = 3, 2%), and *Pseudomonas aeruginosa* (*n* = 3, 2%).

All patients received appropriate antimicrobial therapy. The most frequent antimicrobial regimens administered during the first 24 h of hospitalization were azithromycin and ceftriaxone (17% immunocompromised vs. 31% non-immunocompromised), levofloxacin (16% immunocompromised vs. 14% non-immunocompromised), and levofloxacin and piperacillin–tazobactam (12% immunocompromised vs. 8% non-immunocompromised). Additionally, regimens including vancomycin were prescribed for 39 (42%) immunocompromised and 61 (33%) non-immunocompromised patients.

### 3.3. Severity of Disease

A total of 24 (26%) patients in the immunocompromised group and 43 (23%) in the non-immunocompromised group were admitted to the ICU during hospitalization. Pleural effusion was present in 28 (30%) of immuncompromised patients and 59 (32%) of non-immunocompromised patients. No patients had pulmonary abscesses. In regard to hypoxemia at time of admission, 4 (4%) immunocompromised patients and 16 (9%) non-immunocompromised patients had oxygen saturation less than 88%; 26 (28%) immunocompromised patients and 63 (34%) non-immunocompromised patients had PaO_2_/FiO_2_ less than 300; and 6 (6%) immunocompromised and 5 (3) non-immunocompromised patients had need of invasive mechanical ventilation.

**Figure 2:**
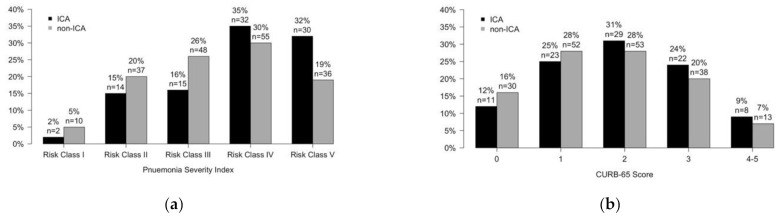
Distribution of severity scores by immunocompromised adults (ICA) and non-immunocompromised adults (non-ICA). Black bars represent ICA, and grey bars represent non-ICA. (**a**) Distribution of Pneumonia Severity Index (PSI) risk classes; (**b**) Distribution of CURB-65 scores.

PSI and CURB-65 were used to quantify severity of CAP and are depicted in Figure 2. A total of 62 (67%) immunocompromised patients were in PSI risk class IV or V, compared to 91 (49%) non-immunocompromised patients. A total of 30 (32%) immunocompromised patients had CURB-65 scores of 3 or higher, compared to 51 (27%) non-immunocompromised patients.

### 3.4. Outcomes

#### 3.4.1. Time to Clinical Stability

**Figure 3:**
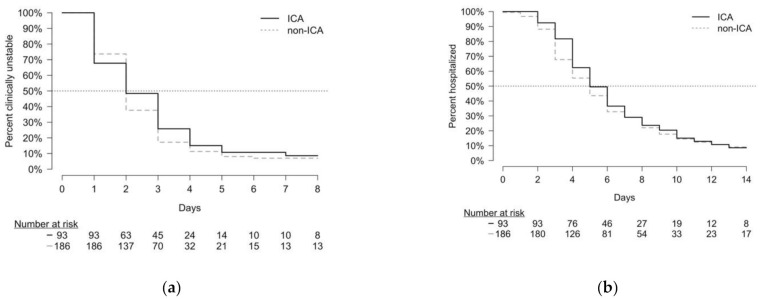
Kaplan–Meier curves for immunocompromised adults (ICA) and non-immunocompromised adults (non-ICA). The black lines represent ICA, and grey dashed lines represent non-ICA. (**a**) Kaplan–Meier curve for time to clinical stability (TCS); (**b**) Kaplan–Meier curve for length of hospitalization (LOH).

The median time to clinical stability was 2 days (IQR: 1–4) among immunocompromised patients and 2 days (IQR: 1–3) among non-immunocompromised patients. A Kaplan–Meier curve for time to clinical stability is shown in Figure 3. In adjusted analysis, immunocompromised and non-immunocompromised matched pairs did not reach clinical stability at different rates (sHR: 0.87; 95% CI: 0.63–1.20, *p* = 0.392).

#### 3.4.2. Length of Hospitalization

Immunocompromised patients had a median length of hospitalization of 5 days (IQR: 4–8), which was also observed in non-immunocompromised patients (IQR: 3–8). A Kaplan–Meier curve for time to hospital discharge is also shown in Figure 3. In adjusted analysis, immunocompromised and non-immunocompromised matched pairs were not discharged from the hospital at different rates (sHR: 0.73; 95% CI: 0.53–1.02, *p* = 0.067).

#### 3.4.3. Clinical Failure

Clinical failure was observed in 4 (4%) immunocompromised patients and 11 (6%) non-immunocompromised patients. After adjustment, the odds of clinical failure were not significantly different between immunocompromised and non-immunocompromised matched pairs (cOR: 0.74; 95% CI: 0.23–2.40, *p* = 0.618).

#### 3.4.4. Cardiovascular Events

A total of 9 (10%) immunocompromised patients experienced a cardiovascular event during hospitalization in comparison to 12 (6%) non-immunocompromised patients. After controlling for co-infection, the odds of experiencing an in-hospital cardiovascular event between immunocompromised and non-immunocompromised matched pairs did not differ significantly (cOR: 1.48; 95% CI; 0.62–3.53, *p* = 0.375).

#### 3.4.5. In-Hospital Mortality

A total of five (5%) immunocompromised patients expired during hospitalization in comparison to five (3%) non-immunocompromised patients. The odds of in-hospital mortality between immunocompromised and non-immunocompromised matched pairs was not significantly different (cOR: 2.00; 95% CI; 0.55–7.33, *p* = 0.296).

#### 3.4.6. Sensitivity Analysis

A total of nine patients in the immunocompromised group had solid advanced cancer with no chemotherapy or other immunocompromising conditions. After removing these patients and matched comparators, there were no statistically significant differences in time to clinical stability (sHR: 0.94; 95% CI: 0.67–1.30, *p* = 0.696), length of hospitalization (sHR: 0.71; 95% CI: 0.5–1.01, *p* = 0.058), clinical failure (cOR: 0.88; 95% CI; 0.25–3.06, *p* = 0.835), cardiovascular events (cOR: 1.59; 95% CI; 0.63–4.04, *p* = 0.326), or in-hospital mortality (cOR: 1.22; 95% CI; 0.22–6.86, *p* = 0.821).

## 4. Discussion

This study indicates that the clinical outcomes of hospitalized patients with CAP due to *Streptococcus pneumoniae* are not different in immunocompromised and non-immunocompromised patients. No differences were observed in the time necessary for patients to reach clinical stability, the length of hospital stay, frequency of clinical failure, frequency of cardiovascular events, or mortality during hospitalization.

Before a patient with *Streptococcus pneumoniae* CAP is admitted to the hospital, the killing of bacteria in the alveoli is dependent primarily on the pulmonary immune response. All patients in this study were treated at time of hospitalization with appropriate antibiotic therapy against *Streptococcus pneumoniae*. Our results suggest that once good antibiotic levels are present in the alveolar space, the killing of *Streptococcus pneumoniae* is produced primarily by the local antibiotic level and is no longer dependent on the local immune response. An abnormal immune system will not alter clinical outcomes in patients with pneumococcal pneumonia who are treated with appropriate antibiotic therapy.

Controversy still exists regarding when a patient with cancer who develops CAP should be considered immunocompromised. In a recent consensus statement regarding the management of CAP in hospitalized immunocompromised patients, it was recommended not to consider patients with early stages of cancer as immunocompromised [1]. In this study, we included only patients with advanced cancer as immunocompromised. We found that advanced stages of cancer and cancer chemotherapy were the most frequent medical conditions and treatments compromising immune function in hospitalized patients with pneumococcal pneumonia. In our sensitivity analysis, we found no differences in conclusions by excluding patients with solid advanced cancer but no other immunocompromising conditions or chemotherapy. Future research in the area is necessary to properly define what patients should be considered at risk from avirulent or opportunistic pathogens.

Severity of CAP at time of hospital admission was evaluated using the PSI, CURB-65, and need of admission to ICU. Even though we expected immunocompromised patients to have more admissions to the ICU, in this study, need for ICU admission was similar for immunocompromised and non-immunocompromised patients. We speculate that immunocompromised patients seek early medical attention once they develop fever or respiratory complaints, and that they have early medical evaluation and early clinical diagnosis. Physicians also have a low threshold for admission and initiation of antibiotic therapy in immunocompromised patients. All these factors may partially explain why immunocompromised patients are not hospitalized with more severe forms of CAP and do not have worse clinical outcomes when compared to non-immunocompromised patients. Differences in PSI risk classes may have been due to the high prevalence of cancer in the immunocompromised group, as neoplastic disease adds 30 points in the PSI score calculation (the largest contributor of any comorbidity). The role of severity scores in immunocompromised patients with pneumonia requires further investigation.

Matching on an appropriately specified propensity score model can yield a set of cases and controls with similar distributions in baseline characteristics [11]. We believe our study achieved this by matching on 21 variables, resulting in low standardized mean differences between groups (all variables matched had standardized mean differences lower than 0.1). With low standardized mean differences, we show that covariate balance between the groups has been demonstrated. Additionally, propensity score matching can be more flexible with cofounder adjustment on outcomes. The “one in ten rule” often followed in regression analysis of dichotomous outcomes would allow a single predictor for our data [12]. Given that only five immunocompromised patients died during hospitalization, in an unmatched analysis of the full cohort, we would not have the flexibility to adjust for additional covariates.

To our knowledge, this is the first study evaluating clinical outcomes in immuno-compromised patients with pneumococcal pneumonia. One major strength of this study was our strict definition of immunocompromising conditions. The weakness of this study was the low number of patients with immunocompromising conditions, with some conditions having very few patients, such as primary immunodeficiency and organ transplantation. Our data may not be generalizable to patients with these conditions. Future studies with a large number of patients may help to describe outcomes for each kind of immunocompromising condition.

## 5. Conclusions

In conclusion, we found that in hospitalized patients with *S. pneumoniae* CAP who are treated with appropriate antibiotic therapy, the presence of an abnormal immune system does influence clinical outcomes.

## Figures and Tables

**Table 1:** Patient characteristics of immunocompromised adult cases and non-immunocompromised adult controls after propensity score matching.

Variable ^1^	ICA (*n* = 93)	Non-ICA (*n* = 186)	SMD ^2^
Age, years (median (IQR ^3^))	63 (56–72)	61 (55–73)	0.021
Male sex	48 (52)	101 (54)	0.054
Black or African American race	19 (20)	42 (23)	0.052
Nursing home resident	6 (6)	10 (5)	0.046
Former smoker	40 (43)	75 (40)	0.055
Obesity ^4^	25 (27)	49 (26)	0.012
Diabetes	22 (24)	46 (25)	0.025
Renal disease	22 (24)	46 (25)	0.025
Coronary artery disease	28 (30)	54 (29)	0.024
Cerebrovascular disease	12 (13)	22 (12)	0.033
Non-cirrhotic liver disease	10 (11)	20 (11)	<0.001
Cirrhosis	2 (2)	3 (2)	0.040
Essential arterial hypertension	56 (60)	115 (62)	0.033
Hyperlipidemia	32 (34)	61 (33)	0.034
Prior myocardial infarction	8 (9)	14 (8)	0.039
Atrial fibrillation	17 (18)	29 (16)	0.072
Chronic antecedent aspirin use	29 (31)	54 (29)	0.047
Hospitalized for >2 days in prior 90 days	30 (32)	53 (28)	0.082
IV antibiotic therapy in the prior 90 days	14 (15)	23 (12)	0.078
Chronic dialysis in the prior 30 days	5 (5)	8 (4)	0.050
Oral antimicrobials in the prior 90 days	22 (24)	42 (23)	0.026

All variables used in this table were matching criteria for propensity score matching. Abbreviations: ICA—immunocompromised adults; non-ICA—non-immunocompromised adults; SMD—standardized mean difference; IQR—interquartile range; IV—intravenous. ^1^ Variables in this table are presented as frequency (percent) unless otherwise noted; ^2^ Standardized mean differences of greater than 0.100 were considered unbalanced after propensity score matching; ^3^ Interquartile range was presented as (25th percentile–75th percentile); ^4^ Obesity was defined as having a BMI greater than or equal to 30.

## Data Availability

The data presented in this study are available upon request from the corresponding author.
